# Optimal Compensation for Temporal Uncertainty in Movement Planning

**DOI:** 10.1371/journal.pcbi.1000130

**Published:** 2008-07-25

**Authors:** Todd E. Hudson, Laurence T. Maloney, Michael S. Landy

**Affiliations:** Department of Psychology and Center for Neural Science, New York University, New York, New York, United States of America; University College London, United Kingdom

## Abstract

Motor control requires the generation of a precise temporal sequence of control signals sent to the skeletal musculature. We describe an experiment that, for good performance, requires human subjects to plan movements taking into account uncertainty in their movement duration and the increase in that uncertainty with increasing movement duration. We do this by rewarding movements performed within a specified time window, and penalizing slower movements in some conditions and faster movements in others. Our results indicate that subjects compensated for their natural duration-dependent temporal uncertainty as well as an overall increase in temporal uncertainty that was imposed experimentally. Their compensation for temporal uncertainty, both the natural duration-dependent and imposed overall components, was nearly optimal in the sense of maximizing expected gain in the task. The motor system is able to model its temporal uncertainty and compensate for that uncertainty so as to optimize the consequences of movement.

## Author Summary

Many recent models of motor planning are based on the idea that the CNS plans movements to minimize “costs” intrinsic to motor performance. A minimum variance model would predict that the motor system plans movements that minimize motor error (as measured by the variance in movement) subject to the constraint that the movement be completed within a specified time limit. A complementary model would predict that the motor system minimizes movement time subject to the constraint that movement variance not exceed a certain fixed threshold. But neither of these models is adequate to predict performance in everyday tasks that include external costs imposed by the environment where good performance requires that the motor system select a tradeoff between speed and accuracy. In driving to the airport to catch a plane, for example, there are very real costs associated with driving too fast and also with being just a bit too late. But the “optimal” tradeoff depends on road conditions and also on how important it is to catch the plane. We examine motor performance in analogous experimental tasks where we impose arbitrary monetary costs on movements that are “late” or “early” and show that humans systematically trade off risk and reward so as to maximize their expected monetary gain.

## Introduction

In the execution of any movement, there is always timing uncertainty. This uncertainty has two major consequences. First, it limits performance on any task for which there are costs associated with temporal imprecision. Second, it has implications for how the motor system should plan movements when the costs of temporal imprecision are asymmetric. In hurrying to catch a subway train, for example, the cost of arriving early is usually small compared to the cost of arriving late and missing the train. An optimal movement planner must take into account temporal reward asymmetries in forming movement plans.

The complexity of movement planning under risk is further increased because temporal uncertainty in the motor system changes constantly. Two major sources of variation in temporal uncertainty occur over different time courses and have different properties: One is a uniform, global shift in temporal uncertainty possibly due to aging, fatigue, injury or disease [1]–[9]. The second is a linear increase in the standard deviation of movement duration with increases in mean movement duration [10].

**Figure 1:**
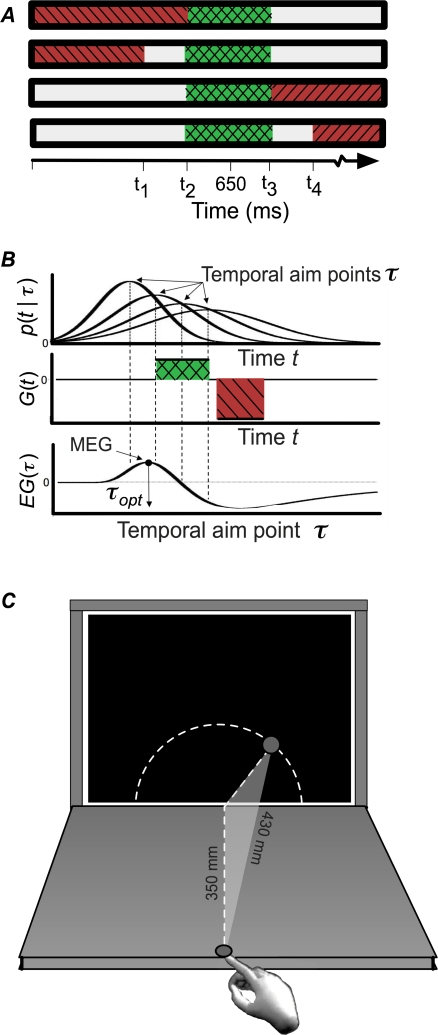
Reward/Penalty Configurations and Expected Gain. (A) Four reward/penalty configurations. The horizontal axis represents time (ms) and the color of each interval specified the reward the subject received if the reach time fell within that interval. Intervals that incurred penalties (−36 points) were coded red (also striped in the figure), those that earned reward (+12 points), green (also cross-hatched). The choice of times *t*_1_,…,*t*_4_ that defined the reward and penalty regions was defined based on each subject's movement duration variance (for a target duration of 650 ms) to equate task difficulty. (B) Expected gain calculation. Upper Panel: The Gaussian distribution of actual movement durations *t* for four choices of planned movement durations *τ*. The vertical dashed lines mark four possible planned movement durations. Standard deviations *σ*(*τ*) increase linearly with planned duration. Middle Panel: The gain *G*(*t*) associated with each actual movement duration *t*. Lower Panel: Expected gain *EG*(*t*) as a function of planned movement duration *τ*. Expected gain is determined by the probability that the actual movement duration falls into the reward or penalty regions. The maximum expected gain (MEG) and the corresponding planned movement duration *τ_opt_* are indicated. (C) Schematic diagram showing the geometric relationship between the start position of the reach and the circular arc along which spatial reach targets were drawn. Reach distance was always 430 mm, regardless of the position of the target along the arc.

Here we use a model of optimal temporal movement planning to investigate the control of movement duration in the face of these two types of temporal uncertainty while human subjects attempted to touch a computer screen within a specified temporal window. We introduced asymmetries in the penalties imposed for early vs. late movement timing (Figure 1A), while at the same time increasing subjects' temporal uncertainty by adding Gaussian noise with 25 ms standard deviation (see Methods). As in all models of motor planning and motor control based on decision theory, we are concerned with the interplay of three elements: possible decisions (here planned movement time, *τ*), uncertainty in the mapping of motor decisions to motor outcomes (represented by the family of probability distributions *p*[*t*|*τ*]), and the costs/benefits resulting from those motor outcomes, *G*(*t*). The mathematical models considered here are part of a growing literature on Bayesian decision models of motor phenomena, such as models of motor adaptation [11]–[13] and motor planning/control e.g., [14]–[21], including the use of prior information in spatial [16],[18] and temporal [17] motor planning, the use of asymmetric cost functions in spatial motor planning [14]–[15],[19] and when selecting a speed-accuracy tradeoff [20]–[21]. The neural computation of decision variables such as those considered here and in previous work has also begun to be investigated [22]–[25].

Figure 1B illustrates the computations needed to maximize expected gain with temporally asymmetric penalties. When discussing movement duration, we must distinguish between the planned arrival time, denoted *τ*, and the actual arrival time, *t*. When movements are executed, the actual arrival time will be unpredictably earlier or later than *τ*. In Figure 1B we show four possible choices of *τ* and outline the calculation of expected gain for each. Note that the optimal planned arrival time need not fall within the temporal reward window.

Human performance will be optimal if the CNS learns its linear temporal uncertainty function,
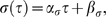
(1)as it relates to planned movement time (*τ*), and uses this information (*α_σ_* and *β_σ_*) to plan reach times that maximize expected gain. Human performance in our task could be sub-optimal in several ways, each depending on the type of information the CNS maintains about Equation 1. We consider 5 such sub-optimal models, denoted *M*_1_, …, *M*_5_. In the first three of these, subjects fail to take account of *α_σ_*, *β_σ_*, or both when planning reaches. In model *M*_1_, subjects fail to compensate for the experimentally imposed static increase in temporal uncertainty due to the added Gaussian noise (SD = 25 ms); in *M*_2_ subjects fail to compensate for the linear increase in temporal uncertainty with increasing reach duration; and in *M_3_* subjects fail in both respects (for details, see Methods: Data Analysis and Model Comparison). Models *M*_4_ and *M*_5_ were analogous to models *M*_2_ and *M*_3_, respectively, but assumed the offset or slope were unknown and hence not fixed to match the training data or added 25 ms timing uncertainty. We compare subjects' performance to each of these sub-optimal movement strategies, and to the optimal strategy (*M*_0_) that results in maximum expected gain.

## Results

### Training

**Figure 2:**
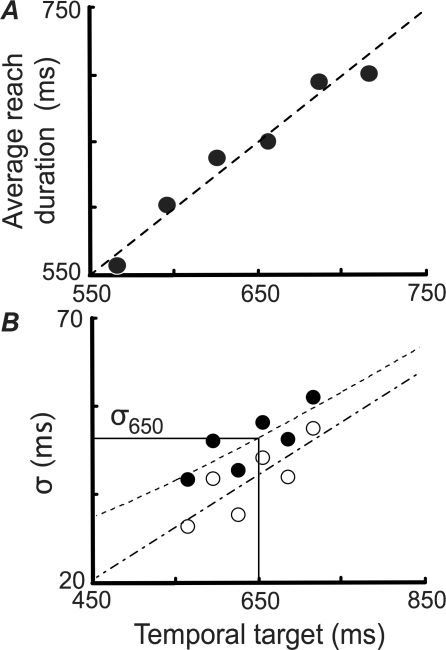
Data from the Training Trials for One Subject (HT). (A) Mean observed time versus experimenter-specified target time with a line of slope = 1, intercept = 0 superimposed. (B) Temporal uncertainty *σ*(*τ*) is plotted as a function of planned movement duration *τ* for both noise-added (filled symbols, dashed line) and unperturbed (open symbols, fitted dash-dotted line) data. The estimated uncertainty *σ*_650_ for a movement of planned duration 650 ms was used to equate the difficulty of the task across subjects. Subjects' fitted slopes (unperturbed) are provided in Figure 3.

**Figure 3:**
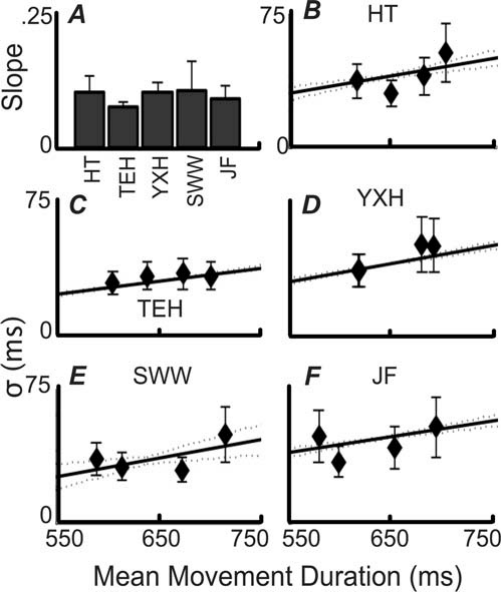
Temporal Uncertainty Functions by Subject. (A) Fitted slope values *α_σ_*±1 SE for temporal uncertainty functions calculated from training data. The corresponding intercepts (*β_σ_*) were −25, −19, −26, −31 and −10 ms, respectively. (B–F) Temporal uncertainty functions calculated from unperturbed training data (solid lines; the dotted lines represent±1 SE) with temporal uncertainty measured during each of the four experimental conditions (diamonds) overlaid.

During training trials, subjects attempted to produce reaches with an experimenter-specified temporal duration; no rewards or penalties were imposed. In Figure 2A, we plot the mean movement duration as a function of the target duration for subject HT. The points lie near the identity line, indicating that the subject could accurately produce a wide range of movement times on command. Figure 2B shows the temporal uncertainty function (the standard deviation of arrival times as a function of target duration, with and without the added noise) measured during training for the same subject. As expected, unperturbed standard deviations (dot-dashed line, open symbols) increase linearly across this range. Estimated Weber-noise parameters (*α_σ_*) for all subjects' temporal uncertainty functions, and verification of the stationarity of those functions (across the training trials and the subsequent main experiment), are provided in Figure 3. Note that fitted functions obtained from training data (lines) and the standard deviations measured during main-experiment reaches (filled diamonds) were well-matched, consistent with the idea that subject performance did not change during the experimental reaches.

### Main Experiment

**Figure 4:**
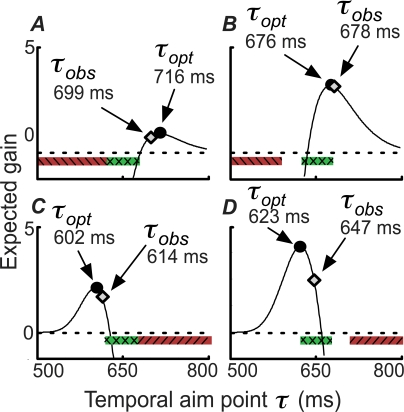
Expected Gain as a Function of Planned Movement Duration for One Subject (HT). The expected gain *EG*(*τ*) for each possible planned movement duration is shown as a solid line. MEG points are marked as circles and observed mean durations are marked as diamonds. The four panels A–D correspond to the four conditions in Figure 1A. Reward and penalty regions are coded as in Figure 1A.

Each of the models makes predictions of reach durations that are based on the aspects of the temporal uncertainty function it incorporates. Because the optimal model (*M*_0_) incorporates both components of the temporal uncertainty function, it can take account of the temporal noise actually experienced by each subject when planning reaches, in turn allowing it to predict optimal movement times. Three of the sub-optimal models (*M*_1_–*M*_3_) each specify only a portion of the actual temporal noise experienced by subjects. Because these models cannot account for the full temporal uncertainty function, their predicted ‘best’ movement times are sub-optimal. For each subject and model, we derived predictions of the mean duration in each of the four conditions that would maximize expected gain in the task given that temporal uncertainty function (see Methods: Model Predictions; Figure 4 illustrates these calculations for an example subject). These predictions allow us to compare observed performance in the task to the theoretical performance of subjects who maximize expected gain under the constraints imposed by each of the four models. In addition to these four models, we considered two sub-optimal models that did not have fixed parameters (*M*_4_ and *M*_5_). In models of this type, the model likelihood (see Method: Data Analysis and Model Comparison) is calculated by integrating over the possible values of the unknown parameters (e.g., overall noise level).

The results of a Bayesian comparison of the performance of the four models (see Methods: Data Analysis and Model Comparison) favored the optimal model *M*_0_ over the sub-optimal models; yielding 11.5 dB in favor of *M*_0_, but −60.5 dB, −11.5 dB and −41.4 dB of evidence for *M*_1_, *M*_2_ and *M*_3_, respectively. Models *M*_4_ and *M*_5_ are less constrained, resulting in evidence below −100 dB. Negative evidence is evidence against a model relative to the other possible models. In our previous work [26] we have used 3 dB evidence, corresponding to odds of nearly 2∶1, as a minimal guideline for inferring an advantage for a model over its competitors. The 11.5 dB evidence for *M*_0_ is strong, corresponding to nearly 15∶1 odds in favor of the optimal model over the set of alternatives.

To assess inter-subject variability, we recomputed the evidence values for 5 subgroups of subjects, with each subgroup consisting of all subjects but one. The change in evidence that occurred as we left each subject out is a measure of how much the conclusions we draw are based on one subject alone. While the evidence decreases somewhat when each subject is removed (and it should since we are basing our conclusion on fewer data), it always favored *M*_0_, and always by at least 7.5 dB, consistent with the conclusions based on all subjects taken together. We note, in particular, that removing the non-naive subject who was an author (TEH) still resulted in evidence of 9 dB in favor of *M*_0_.

**Figure 5:**
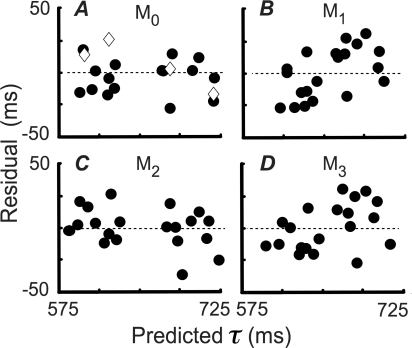
Residuals. Residual differences between mean movement duration and model predictions under the assumptions of each of the four models (*M*_0_–*M*_3_). Data from HT, as described in Figure 4, are plotted as diamonds.

In addition, we plotted, for all subjects and conditions, the mean observed movement duration as a function of the duration predicted by each of the four models (Figure 5 plots the deviations of the actual from the predicted movement times). In such a plot, consistency of the data with the model corresponds to the data falling along the identity line. We computed linear regressions of observed mean duration as a function of predicted mean duration for each of the four models. Only *M*_0_ had a best-fit slope and intercept whose confidence intervals contained those of the identity line (Table 1), corroborating the result of the Bayesian model comparison. We conclude that the evidence favoring *M*_0_ over any of the competing models is overwhelming, implying that subjects compensated for their increased uncertainty at longer durations and also for the 25 ms added uncertainty imposed experimentally.

**Table 1:** Linear Fits of *t_obs_* to *t_opt_* and 95% Confidence Bounds.

Model	*α_t_*	*β_t_*
*M_0_*	0.94, **[0.85:1.03]**	41 ms, **[−20:101]**
*M_1_*	1.34, [1.18:1.49]	−223 ms, [−325: −124]
*M_2_*	0.89, [0.81:0.97]	71 ms, [17:123]
*M_3_*	1.2, [1.05:1.35]	−143 ms, [−243: −46]

Confidence intervals in bold span the relevant parameter value of an identity line.

To investigate how the suboptimal models fail, we present differences between observed average temporal endpoints and model predictions for each of the four models (Figure 5). For each of the sub-optimal models, we describe how the pattern would appear if data were fit with that model.

Model *M*_1_ compensates for increased temporal uncertainty with increased movement duration but fails to compensate for the *σ* = 25 ms temporal noise added experimentally. Subjects conforming to this model will have temporal aim points closer to the center of the target region than they should be since they are based on an erroneously small estimate of temporal uncertainty. That is, compared with the optimal model (*M*_0_), model *M*_1_ predicts longer durations for predictions of durations shorter than the target duration (650 ms), and shorter durations for predictions longer than the target duration. Thus, we predict the left-hand cloud of residuals to move down and right and the right-hand cloud to move up and left, which is precisely what happened (upper-right panel, Figure 5).

Subjects employing model *M*_2_ (lower-left panel, Figure 5) would fail to take duration-dependent noise into account, but compensate for the *s* = 25 ms temporal noise added experimentally. Such subjects overestimate noise for short durations and underestimate it for long durations. Intuitively, the residuals should move up and left. This is true of most data points, but not all. The intuitive pattern is occasionally broken due to the complex, nonlinear calculation of expected gain (Figure 1B) and the switch from the veridical uncertainty function (*M*_0_) to an incorrect, flat function (*M*_2_). As expected, the predictions of *M*_3_ combine the shifts of the other two suboptimal models.

In summary, based on the comparison of the optimal and three suboptimal models, we conclude that subjects delayed or advanced their temporal endpoints in accordance with the calculated optimal times defined by *M*_0_. The Bayesian model comparison employed is novel and correct for comparison of non-nested models (see Method: Data Analysis and Model Comparison). We also carried out a set of statistical tests based on linear regression of actual versus predicted times. The conclusions based on these regressions tests are identical to those just reported: we reject models *M*_1_, *M*_2_ and *M*_3_ but not *M*_0_ (Table 1).

The gains earned by subjects potentially provide an additional dimension for testing the models. We have compared actual gains to expected gains predicted by each of the models. However, the gain functions are flat relative to the sampling variability of observed points earned, so that this analysis does not serve to differentiate the models.

### Learning

To investigate the possibility that subjects used a hill-climbing strategy during the main experiment, instead of maximizing expected gain by taking account of their own temporal uncertainty function and experimentally imposed gain function, we performed a hill-climbing simulation using each subject's temporal uncertainty function. In the simulation, intended duration was moved away from the penalty region by 3Δ*t* ms after each penalty and towards the center of the target region by Δ*t* ms for each miss of the target that occurred on the opposite side from the penalty (corresponding to the 3∶1 ratio of penalty to reward). The value of Δ*t* was initially set to be relatively large. With each change of direction of step, Δ*t* was reduced by 25% to a minimum step size of 1.5 ms. While this simulation approximately reproduced the final average reach times observed experimentally, it does not provide a good model of subject performance. First, there were significant autocorrelations of reach durations beyond lag zero in the simulation data but not in the experimental data. Second, a learning algorithm would be expected to produce substantially higher *σ* values during test than those observed during training. This is what we found with our hill-climbing simulation. Using subjects' training *σ* values to produce the simulated data, the simulation produced 17 out of 20 main-experiment *σ* values that were above the training values, whereas our subjects' main-experiment *σ* values (Figure 3) were entirely consistent with temporal uncertainty functions measured during training.

## Discussion

### Movement Planning as Gain Maximization

To move accurately, an organism's motor system must generate an intricate series of precisely timed neural commands. The exact nature of these commands is not known. Whatever the format of the command signals [27]–[32], movement controlled by any physical controller-actuator system, including biological motor systems, will always exhibit some motor uncertainty. Nevertheless, it is possible to plan movements that will maximize expected gain in the face of that uncertainty. To do so, an organism must be capable of assessing both the probabilities of possible movement outcomes and their consequences.

One of the most thoroughly studied cases in which humans integrate the probabilities of possible movement outcomes and their consequences is the tradeoff between movement speed and spatial accuracy [20]–[21], [33]–[34]. However, in our experiment we were concerned with temporal accuracy, and faster movements are typically more temporally accurate (the opposite of the spatial speed-accuracy tradeoff). By imposing costs for early/late arrivals, we were able to determine whether the motor system is capable of picking movement times that maximize expected gain, taking into account temporal uncertainty.

We conclude that, in the timing task we examined, the motor system estimates and compensates almost perfectly for its own temporal uncertainty and correctly anticipates how that uncertainty interacts with the asymmetric reward structure of the environment. This outcome is plausible given the close neurophysiological links between motor timing and the assessment of probabilities and consequences [22]–[25], [35]–[37].

We note however that it has been argued that a representation of time plays no role in one of the most basic forms of motor learning: motor adaptation [38]. The current study provides evidence that the motor system is capable of using a representation of time in at least some circumstances where the consequences of the movement are unambiguously linked to the timing of the movement, and in addition that it does so optimally.

### Timing as an Element of Movement Optimization

Several models of spatio-temporal movement control are based on optimizing an internal cost function that either includes or predicts movement timing. One such model of trajectory formation, the *minimum variance model* [39], assumes that the CNS selects a spatio-temporal reach trajectory by optimizing a cost function based on the movement's endpoint variance. In particular, the minimum variance model selects “…the temporal profile of the neural command … so as to minimize the final positional variance for a specified movement duration…” [39], p. 782. More recently the *minimum-time model* of trajectory formation has been proposed [40] based on the assumption that, subject to a constraint on movement accuracy, the CNS attempts to minimize movement duration. In both models, the speed-accuracy tradeoff is modeled by scaling the spatial variance of the reach with the amplitude of the motor control signal; that is, they assume signal-dependent spatial motor noise.

In the absence of signal-dependent noise, both models would predict a ‘bang-bang’ control scheme, where the control signal takes first a maximum positive and then maximum negative value producing alternating maximum forward and reverse accelerations leading to maximum movement speed and hence minimum duration. However, bang-bang control predicts trajectories that are inconsistent with typical motor behavior. By modeling spatial noise as signal-dependent, it is possible to predict a range of important behavioral results with both the minimum-variance and minimum-time models, such as the smooth variation in spatial and temporal reach profiles e.g., [41]–[42], Fitts' law [33], and the spatio-temporal details of saccadic trajectories [43].

Unlike these previous studies, here the emphasis is on accuracy of movement duration. This results in a reverse speed-accuracy tradeoff; slower movements have lower temporal accuracy (even though they have higher spatial accuracy). We show that, in a task where spatial uncertainty (and therefore signal-dependent spatial noise) plays essentially no role, reach durations are selected to nearly maximize expected gain in the presence of duration-dependent temporal uncertainty.

Duration-dependent temporal uncertainty constitutes a constraint on the temporal aspects of movement planning that is similar in many respects to the planning constraint imposed by signal-dependent spatial noise. Simultaneously minimizing temporal and spatial noise provides a method of solving the underconstrained problem of trajectory selection. Although several previous studies have proposed multiply-constrained models of movement planning [44]–[45] and the duration-dependence of temporal uncertainty is well known e.g.,[10]; [46]–[47], we provide the first demonstration of the CNS making use of its own temporal uncertainty in movement planning. While selecting the movement trajectory that minimizes spatial and/or temporal noise is a possible method of movement planning, the optimal movement planner carefully separates the constraints imposed on spatial and temporal accuracy (duration-dependent temporal noise and signal-dependent spatial noise) with the costs of spatial and temporal errors, which we discuss next.

### Cost Functions in Models of Movement Planning

In both the minimum-time and minimum-variance models [39]–[40], a trajectory is selected so as to optimize an internal cost for spatial variance or movement duration (respectively) in the presence of signal-dependent spatial noise. The cost is internal in the sense that it does not make reference to any externally imposed costs on movement errors, such as monetary rewards and penalties that may be imposed due to one's spatial precision or movement duration. There have been a large number of models of movement based on the optimization of internal cost functions that identify movement cost with an invariant kinematic or dynamic variable (time [48], spatial precision [39], torque-change [49]–[50], jerk [51], etc.). However, there are pitfalls inherent in identifying movement cost with an aspect of the movement itself, despite the current movement goals. For example, the minimum-variance model always chooses a movement with the best possible spatial precision, even when that level of precision is unnecessary for the task. Similarly, the minimum-time model always chooses the shortest duration movement that satisfies the constraint on spatial precision even when, as in some conditions of the current study, an external temporal cost function rewards longer-duration movements.

Recent models of optimal movement planning e.g., [14],[18],[26],[44] approach the problem somewhat differently. In these models, which have previously been used to predict spatial movement endpoints [14],[18] and movement trajectories [44], the difference between a constraint on movement planning and a cost incurred from movement error must be recognized. While duration-dependent temporal noise, signal-dependent spatial noise, energy consumption, biomechanics, etc. constitute constraints on movement planning and control, they are not properly costs. A cost essentially imposes a weighting on the available constraints, and is task dependent. By experimentally imposing costs [14]–[15], [18]–[21],[26] on spatial or temporal inaccuracy, it is possible to predict flexible movement strategies that incorporate task-relevant constraints (e.g., duration-dependent temporal uncertainty) while effectively ignoring (down-weighting) constraints that are not as important to the task at hand (signal-dependent spatial uncertainty). In the present study, we manipulated the temporal cost function by imposing penalties on too-short reach durations in some conditions, and too-long durations in other conditions, and determined whether subjects responded appropriately to these different cost functions.

We have modeled movement planning as minimizing an external gain function in the presence of task-relevant internal temporal noise. By identifying the to-be-minimized cost with the movement goal we have separated fixed kinematic/dynamic variables from the purpose of the movement. This allows us to predict flexible movement plans that may minimize spatial or temporal uncertainty, but only when that is relevant to the task at hand. A deeper understanding of movement planning and execution will result from models that similarly separate cost functions from fixed sets of kinematic/dynamic variables while simultaneously taking account of task-relevant spatial and/or temporal uncertainty.

## Materials and Methods

Subjects were instructed to reach to a computer screen. Prior to each reach, a timer bar was presented on-screen, indicating the timing of the rewarded and penalized temporal windows, along with a circular spatial target. To earn rewards, subjects had to touch within the circular target area within a specified temporal window (“temporal target”). All spatial targets (12 mm radius) were presented along a circular arc 430 mm from the start position (Figure 1C). The timer bar was used to indicate the reward structure of each trial (described below), and also to signal to the subject the movement duration achieved following completion of each reach. All measurements (spatial and temporal) were made with an Optotrak 3020, sampling at 200 Hz. Reach initiation was defined as the moment when the fingertip moved (at least) 2 mm toward the computer monitor, and reach termination as the time when the fingertip arrived within 3 mm of the monitor and the forward fingertip velocity fell below 3 mm/s. Subjects were seated facing the center of the (upright) computer monitor.

The start position of the reach was on the tabletop, in front of the upright computer screen. Fingertip position was controlled at the start of each reach, and constrained to be within 1 mm of the start position. The start position was 350 mm in front of the center of the monitor's bottom edge (Figure 1C). Target locations were selected from a circular arc on the screen. The arc was centered on the projection of the start position to the bottom edge of the screen (Figure 1C). All points on this arc were equidistant from the start position. Reaches were made in a dimly lit room (the majority of the light coming from the CRT), and subjects could see their hands. No feedback was presented on the screen showing the fingertip landing point, although an auditory beep indicated that the target had been touched.

Subjects were not told that Gaussian noise with *σ* = 25 ms was added to *all* measured temporal endpoints. This added noise, in combination with subjects' natural duration-dependent variations in temporal uncertainty, allowed us to determine whether subjects were sensitive to changes in the two sources of variation in temporal uncertainty described above. The noise-added temporal endpoint was displayed after each reach, shown as a thin line intersecting the timer bar at the appropriate position.

Each subject completed two sessions, a training session and the main experiment. Both sessions were completed within the same hour on a single day.

### Training

Subjects were first given a training session in which temporal targets (width: 3 ms, no adjacent penalty region) were presented at six target durations (565, 595, 625, 655, 685 and 715 ms; 8 repetitions each, in separate blocks, followed by 50 repetitions each, in separate blocks) spanning the range of temporal aim points observed during pilot work. Although this window was too narrow for subjects to reliably hit, subjects were not scored during training, and were told simply to time their reaches as closely to each target time as possible. This session allowed us to estimate the standard deviation of each subject's movement durations for a set of precisely known target durations, and also allowed subjects to learn their own (noise-added) temporal uncertainties in the task. Standard deviations at each target time (Figures 2B and 3) were measured from the final 40 repetitions to avoid possible initial practice effects.

### Main Experiment

Immediately following training, subjects were given a temporal target centered at 650 ms, with a half-width of 0.6*σ*_650_, where *σ*_650_ was the estimated SD of movement duration for a mean duration of 650 ms. In this way, we equated the difficulty of the task across subjects based on their training performance.

Subjects were paid a bonus for touching the spatial target within the temporal target window (Figure 1A, green, cross-hatched bars), and penalized for touching the spatial target within a temporal penalty window (Figure 1A, red, striped bars) or for failing to touch the spatial target. Four blocked conditions were employed (Figure 1A), two early temporal penalty conditions and two late penalty conditions (64 trials each). The two early temporal penalty regions began at 0 ms and ended either 0.6*σ*_650_ or 1.35*σ*_650_ ms prior to 650 ms. The two late temporal penalty regions began either 0.6*σ*_650_ or 1.35*σ*_650_ ms following 650 ms, and were open-ended.

The outcome of each trial was signaled by distinct auditory tones notifying the subject that a reward was earned or a penalty assessed. The possible reward earned on any trial was $0.12 and the penalty was −$0.36 (or −$0.60 for missed spatial targets). Note that the ratio of penalty to bonus magnitudes was 3∶1. Trials in which the spatial target was not touched were re-run (fewer than 1% of all trials) to equate the number of touched-target trials in each condition. The untouched-target trials were not analyzed.

### Subjects

Subjects were four students at New York University who were not aware of the purpose of the experiment and one author (TEH). All subjects gave informed consent before the experiment. The experimental protocol had been approved by the Institutional Review Board at New York University.

### Model Predictions

As described in the Introduction, decision theoretic models of motor behavior are concerned with the interplay of three elements: movement strategy, uncertainty, and the gain or loss from possible movement outcomes. The interplay of these three elements is represented graphically in Figure 1B for the optimal model, *M*_0_. Calculation of the temporal endpoints predicted by each of the models to be considered required that the expected gain, in terms of average bonus earned per reach, be computed based on the constraints supplied by the hypothetical system. For example, the optimal neuromotor controller would make use of information concerning both Weber-like increases in temporal uncertainty with increasing reach time, and the experimentally increased overall temporal uncertainty.

A given motor strategy or plan, ***s***, determines the critical states of the system. Although motor plans are complex sequences of control signals in time, the only consequence of the choice of motor plan in our task is to select an expected temporal endpoint, *τ* ***_s_***. The expected gain from ***s*** is then given by (Figure 1B):
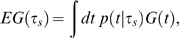
(2)where *G*(*t*) describes the gain or loss associated with a particular temporal endpoint (Figure 1A and Figure 1B, middle panel). The term *p*(*t* | *τ_s_*) describes the probability density of temporal endpoints expected from any chosen movement strategy ***s***. Note that these are planned durations, not reaction times, and hence we have no *a priori* expectation that these distributions will be skewed. We model the duration distribution as a Gaussian with mean arrival time *τ_s_* and a standard deviation *σ*(*τ_s_*)
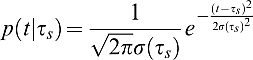
(3)(QQ plots of these distributions confirm that the Gaussian distribution models the data well). The temporal uncertainty function, *σ*(*τ_s_*) is able to capture the well-known Weber-like scaling of temporal standard deviation with mean arrival time *τ_s_* (Figure 1B, top panel). We used values estimated from each subject's training data to compute individual *σ*(*τ_s_*) functions for models *M*_0_–*M*_3_.

In Figure 1B (bottom panel), for the rightmost choice of *τ*, the probability of arrival in the penalty zone is nearly as high as that of arrival in the reward zone. This choice of *τ* is likely to lead to nearly as many penalties as rewards. Given that the penalty/reward ratio was 3∶1, expected gain is negative for this choice of *τ*. The distribution associated with the leftmost choice of *τ* is primarily in the uncolored time zone where the subject earns nothing. This choice of *τ* is likely to lead to rare rewards and extremely rare penalties, resulting in only a small total reward across many trials. Interestingly, a third choice of *τ*, centered on the temporal reward region, earns even less than the previous choice of *τ* because of a combination of its proximity to the temporal penalty, the magnitude of temporal movement noise, and the ratio of the reward to penalty magnitudes.

The best of the four choices shown is therefore the *τ* located at the left edge of the rewarded temporal region. Of the four shown, it makes the best compromise between the width of the probability distribution for *t* and its distance from the centers of the reward and penalty regions, given the widths of those regions and the ratio of gains to losses. Of course, there are infinitely many possible choices of *τ*. The lower panel shows the expected gain as a function of *τ*, with the maximum expected gain (MEG) point indicated with a circle at the peak of the expected gain function. If observers select this value *τ_opt_*, they maximize their expected gain.

We computed *τ_opt_* for each of the four penalty conditions and each subject based on an estimated temporal uncertainty function *σ*(*τ_s_*) that was specific to each subject. In all cases the optimal (maximum expected gain) value of *τ_s_* was shifted away from the penalty region.

### Data Analysis and Model Comparison

The optimal Bayesian model (*M*_0_) makes full use of the temporal uncertainty function *σ*(*τ_s_*) from each subject's training session. The five sub-optimal models use less information. *M*_1_ uses the *σ*(*τ_s_*) calculated from each subject's training data without the experimentally added *σ* = 25 ms noise. *M*_2_ uses each subject's constant *σ* for all *τ_s_* that includes the overall added *σ* = 25 ms noise; it uses the square root of the average of perturbed variances about the target durations measured during training. *M*_3_ uses the subject's constant *σ* without the experimentally added noise. *M*_4_ and *M*_5_ use a constant offset and constant offset and slope, respectively, but assume that the values of these parameters are unknown. Of course, some subjects are more accurate than others but this is explicitly taken account of in our analysis. Each model's predictions are defined in terms of performance relative to an individual's temporal uncertainty function. Subjects who are inherently poorer timers are being compared to a standard (defined by each model) that is tailored to (defined in terms of) the limits of that subject's abilities. So while there are in fact individual differences between subjects, these were removed in the design and analysis of the experiment. Because we equated subjects in this way we could analyze group data.

The predicted movement strategy, *s*, is therefore a function of the type(s) of temporal uncertainty information incorporated by each model *M_m_*, the reward structure defined by the *j* ^th^ experimental condition (*j* = 1 to 4), and the temporal uncertainties measured during training for the *k* ^th^ subject (*k* = 1 to 5). Let 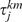 denote the value of *τ* predicted by model *M_m_* based on an estimate of timing uncertainty calculated from the assumptions of each model. For convenience, we denote the temporal uncertainty for an attempt to produce a movement duration of 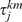 (using the full temporal uncertainty function based on the training trials), 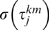, as 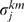.

The models we considered are not all nested and consequently we chose a method of model comparison for non-nested models [52]–[54] that we describe next. Let 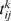 denote the *i* ^th^ arrival time (of the 64 trials per condition) in condition *j* for the *k* ^th^ subject. The likelihood of model *M_m_* is given by:
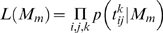
(4)where
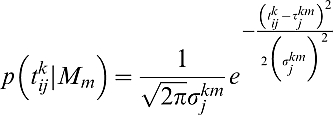
. (5)Note however that for *M_4_* and *M_5_*, the model likelihood must be calculated by integrating over the unknown parameters: the constant offset, 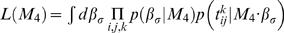, and constant offset and slope, 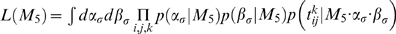, of the temporal uncertainty function, respectively, where the prior probability distributions over the parameters are taken to be bounded Jeffreys (uninformative) priors [55].

Let *π*(*M_m_*) denote the prior probability of the *m* ^th^ model. Then the posterior probability of the *m* ^th^ model given the data is
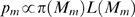
(6)and
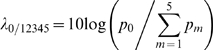
(7)is a comparison of the posterior probability of the optimal model *M*_0_ to the combined posterior probabilities of sub-optimal models: it is a *measure of evidence* [53] favoring the optimal model (the factor of 10 allows us to express evidence in decibels, denoted dB). A similar evidence measure can be computed for each of the sub-optimal models using the odds ratio of the probability of each sub-optimal model to the combined probability for the remaining five models (four sub-optimal and one optimal). We set the prior probabilities of the six models to be equal and computed these evidence measures.
